# Neurophysiology of the cerebellum and clinical correlations: a review

**DOI:** 10.1055/s-0045-1811623

**Published:** 2025-09-19

**Authors:** Walderico Silva Machado Filho, Alberto Rolim Muro Martinez, Marcondes Cavalcante França Junior

**Affiliations:** 1 Universidade de Campinas, Faculdade de Ciências Médicas, Departamento de Neurologia, Campinas SP, Brazil.

**Keywords:** Neurophysiology, Cerebellum, Cognition, Cerebellar Ataxia

## Abstract

The cerebellum is a complex structure tightly connected to the cerebral cortex, brainstem, and spinal cord. It plays an important role in movement coordination and motor planning. Lately, it has been also recognized as a key component in cognitive circuits. The specific motor functions of the cerebellum include the control of the initiation, execution, and velocity of movements, as well as the maintenance of balance, motor coordination, and muscle tonus. Cerebellar lesions typically result in ataxia but can also lead to other manifestations, such as abnormal eye movements, severe vertigo, or impaired motor learning. Additionally, the cerebellum plays a key role in cognitive processes, with dysfunction leading to conditions such as the cerebellar cognitive affective syndrome (CCAS). Functional distinctions are evident between the cerebellar vermis, hemispheres, and flocculonodular lobe, each contributing to different motor and cognitive domains. In the current review, we address the current understanding of cerebellar anatomy and physiology, highlighting the most relevant correlations between lesion location and clinical symptomatology for practicing neurologists.

## INTRODUCTION

The cerebellum is located in the posterior fossa, just behind the brainstem. It is often referred to as the “little brain” due to its architectural similarity to the cerebrum, extensive neuronal networks, and intricate connections. It represents only 10% of the total brain volume, but contains a disproportionately high number of neurons, the most relevant of which are known as Purkinje cells. 1 The cerebellum is composed of gray and white matter, with the four deep cerebellar nuclei (fastigial, globose, emboliform, and dentate) embedded within the white matter. This organ takes part in many internal and external circuits, forming complex and essential connections that are fundamental to human function.

Traditionally, the primary role of the cerebellum has been associated with motor control, particularly in regulating movement coordination, velocity, gait, and muscle tone. However, it is now clear that it is also involved in cognitive processes. 2 Indeed, neuropsychological deficits and behavioral abnormalities may arise in patients with cerebellar damage, depending on the location of the lesions. Such deficits may affect executive functions, visuospatial processing, mood regulation, and language. 3

In the following sections of this manuscript, we will review details on the cerebellar cytoarchitecture, functional subdivisions, connections, and clinical correlations between lesion location and clinical symptomatology.

## CEREBELLAR CYTOARCHITECTURE

**Figure 1:**
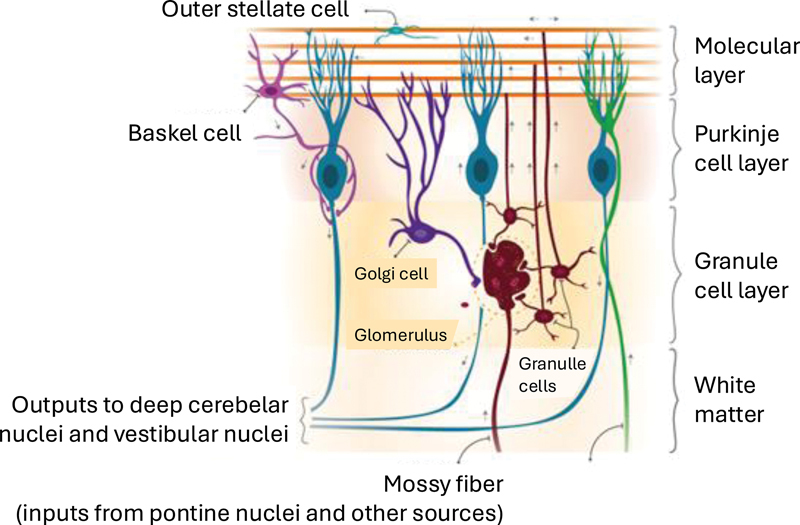
Microscopic circuitry of the cerebellar cortex.

The cerebellum is classically divided into white and gray matter, with the latter including not only the cerebellar cortex (which contains the highest concentration of neuronal cell bodies) but also the deep cerebellar nuclei ( Figure 1 ). Externally, there is the cerebellar cortex, which contains numerous cells, with special emphasis on the Purkinje cells, the largest and most important neurons of the cerebellum.

These highly specialized neurons are now known to be the primary site of damage in many neurogenetic disorders, including spinocerebellar ataxias. Such vulnerability arises from their high metabolic demand, complex morphological architecture, and reliance on efficient mechanisms for DNA repair and ionic homeostasis. Mutations in genes involved in ion channel regulation, calcium metabolism, chromatin organization, and synaptic maintenance are commonly observed in hereditary ataxias. These directly compromise the function and viability of Purkinje cells. 4

The cerebellar cortex is organized into three distinct layers, from superficial to deep: molecular, Purkinje cell, and granular. The molecular layer contains two main types of inhibitory interneurons, basket and stellate cells, as well as parallel fibers, which originate from granule cells. 5 6 The Purkinje cell layer consists predominantly of Purkinje cell bodies, along with dendrites from neurons in adjacent layers. The granular layer is the thickest of the three and contains the highest number of cells, fibers, and synaptic connections. Key components of this layer include granule cells, Golgi cells, mossy fibers, and climbing fibers. Climbing fibers are particularly important, as they transmit excitatory input from the inferior olivary nucleus and vestibular nuclei.

Additionally, the inhibitory basket, stellate, and Golgi cells play a fundamental role in regulating neuronal activity. Golgi cells modulate sensory input and synaptic plasticity within the granular layer, while basket and stellate cells refine Purkinje cell output through somatic and dendritic inhibition, respectively. The coordinated action of these interneurons is essential for the temporal and spatial precision of cerebellar circuits, supporting both motor execution and adaptive learning ( Figure 1 ).

The white matter of the cerebellum is primarily composed of Purkinje cell axons, which synapse on four pairs of deep nuclei, located in the cerebellar interior. From lateral to medial, these are the dentate, emboliform, globose, and fastigial nuclei. The emboliform and globose nuclei, due to their functional similarities, are collectively referred to as interposed. These deep nuclei serve as the primary efferent pathways of the cerebellum. 7

An important cell that also deserves attention is microglia, which resides in the central nervous system, located in both the white and gray matter of the cerebellum. Unlike microglia in other brain regions, the cerebellar ones exhibit distinct morphological, functional, and transcriptomic characteristics. They play a critical role in regulating cerebellar synapses, as well as in the development and maintenance of neuronal circuits—particularly in the maturation of connections between climbing fibers and Purkinje cells, with which they maintain dynamic and specific interactions. In addition to supporting synaptic homeostasis and plasticity, cerebellar microglia display a more reactive immune profile, which may contribute to increased vulnerability in inflammatory and neurodegenerative conditions, such as hereditary ataxias. 8

## FUNCTIONAL ORGANIZATION OF THE CEREBELLUM

The functional organization of the cerebellum is closely related to the anatomy of its lobes and their connections with the deep nuclei within the white matter. It can be divided into the lateral, medial, and intermediate zones, as well as the flocculonodular lobe.

Based on this, functionally, the cerebellum is classified into: spinocerebellum, corresponding to the medial and intermediate zones, primarily involved in motor execution and coordination; cerebrocerebellum, corresponding to the lateral zones, responsible for motor planning and cognitive functions; and vestibulocerebellum, corresponding to the flocculonodular lobe, mainly involved in balance and eye movement control. 6 9

Each functional division establishes direct connections with the deep cerebellar nuclei and the vestibular nuclei via Purkinje cell axons. It is important to highlight that, although the vestibular nuclei are anatomically located in the brainstem, their function is as crucial as the deep cerebellar nuclei. The Purkinje cell axons from the lateral zone project to the dentate nucleus, the axons from the medial zone project to the lateral vestibular and fastigial nuclei, and those from the intermediate zone project to the interposed nucleus. Additionally, Purkinje cells in the flocculonodular lobe project to the fastigial nucleus or directly to the vestibular nuclei. 10

## CIRCUITS OF THE CEREBELLUM

The cerebellum is known for its complex and extensive connectivity with various brain regions, playing crucial roles in both motor and nonmotor functions. The primary output of the cerebellar cortex occurs through Purkinje cells, which synapse with neurons in the cerebellar nuclei. These nuclei, in turn, project to several brain regions, including the brainstem, thalamus, and cerebral cortex, facilitating multimodal integration and motor coordination. Prior to the external afferent and efferent connections, there is a microcircuit among neurons, known as the Internal Circuit of the Cerebellum. 11 12

It is essential to understand two key concepts: inhibitory and excitatory synapses. The type of neurotransmitter released determines the synapse type. In summary, excitatory synapses primarily release glutamate, whereas inhibitory synapses mainly release gamma-aminobutyric acid (GABA), although other neurotransmitters such as glycine, taurine, and acetylcholine may also be involved to a smaller extent. 13 As previously mentioned, the cerebellar cortex is divided into three layers, each containing critical structures for the internal neuronal circuit ( Figure 1 ). Different types of cells form synapses in each layer. The pathway begins with input fibers and ends with the inhibition of the cerebellar nuclei by Purkinje cells.

The input pathways of the cerebellar internal circuit are composed of mossy and climbing fibers, which carry external information and have distinct origins. Climbing fibers derive their name from their characteristic trajectory, as they wrap around the dendrites of Purkinje cells. They originate from the inferior olivary nucleus in the medulla, which receives input from the premotor cortex, brainstem nuclei, and spinal cord, playing a crucial role in motor coordination, control, and learning. 14 After leaving the inferior olivary nucleus, climbing fibers travel through the cerebellar white matter, forming synapses with the deep nuclei before reaching the molecular layer, where they terminate in excitatory synapses with Purkinje cell dendrites.

Mossy fibers primarily originate from the forebrain, brainstem, and spinal cord. Upon entering the cerebellum, they form excitatory synapses with the deep cerebellar nuclei before projecting to the cortex, where they terminate by synapsing with the dendrites of granule cells. Granule cells, in turn, extend their axons into the molecular layer, where they give rise to parallel fibers, which establish excitatory synapses with Purkinje cells.

The internal circuit of the cerebellum also includes several other important cellular components, such as basket, stellate, and Golgi cells. These are inhibitory interneurons that primarily target Purkinje and granule cells, modulating their activity. Most of these inhibitory synapses occur within the granular layer, where the complex network of synaptic interactions among the cells forms a distinctive microscopic structure known as the cerebellar glomerulus. 15

**Figure 2:**
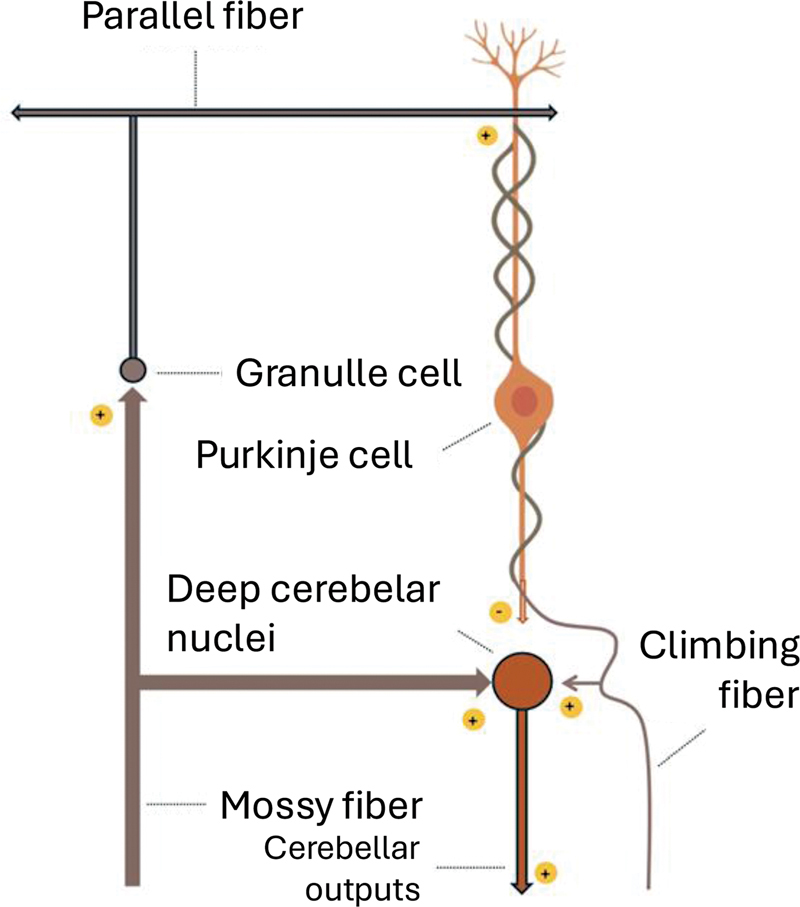
Excitatory and inhibitory synapses of the cerebellar intrinsic circuit.

The cerebellar circuit is far more intricate than described here. In summary, inhibitory synapses are formed by Purkinje, basket, stellate, and Golgi cells. Excitatory synapses are established by climbing fibers, mossy fibers, and granule cells ( Figure 2 ). After processing through this complex network, information is transmitted to the deep cerebellar nuclei, which serve as the primary output structures of the cerebellum.

Many cerebellar disorders share a common pathophysiological mechanism, characterized by an imbalance between inhibitory and excitatory synaptic activity. Growing evidence indicates that transcranial magnetic stimulation (TMS) applied to the cerebellum can modulate neuronal excitability and cerebellar synaptic transmission, as well as influence cerebellocortical and descending motor circuits. These effects are dependent on stimulation parameters such as frequency and pattern, as well as the functional connectivity of the cerebellum. In vitro studies demonstrate that low-frequency stimulation (1 Hz) induces inhibition, whereas high-frequency stimulation (20 Hz) promotes excitation, particularly affecting synaptic transmission in interneurons and Purkinje cells. Additionally, cerebellar TMS modulates spinal reflexes, suggesting an impact on descending pathways such as the vestibulospinal and reticulospinal tracts. Therefore, cerebellar TMS acts as a dynamic modulator of synaptic plasticity and network excitability within motor circuits. 16

## CEREBELLAR OUTPUT PATHWAYS

In the study of cerebellar pathways, it is crucial to understand the functional divisions of the cerebellum and their connections with the deep nuclei. From lateral to medial, these nuclei include the dentate, interposed, and fastigial nucleus, while the flocculonodular lobe lies outside the cerebellar white matter.

The lateral cerebellum, known as the cerebrocerebellum, projects information through Purkinje cells to the dentate nucleus, forming two major pathways.

First is the thalamic fasciculus: after exiting the dentate nucleus, projections decussate at the superior cerebellar peduncle and travel to the ventrolateral thalamic nucleus. From there, they project to the premotor cortex, supplementary motor area, and parietal lobe, playing a critical role in motor planning and integration within the pyramidal tract. Additionally, this cerebellar pathway contributes significantly to executive cognitive functions. 17

**Figure 3:**
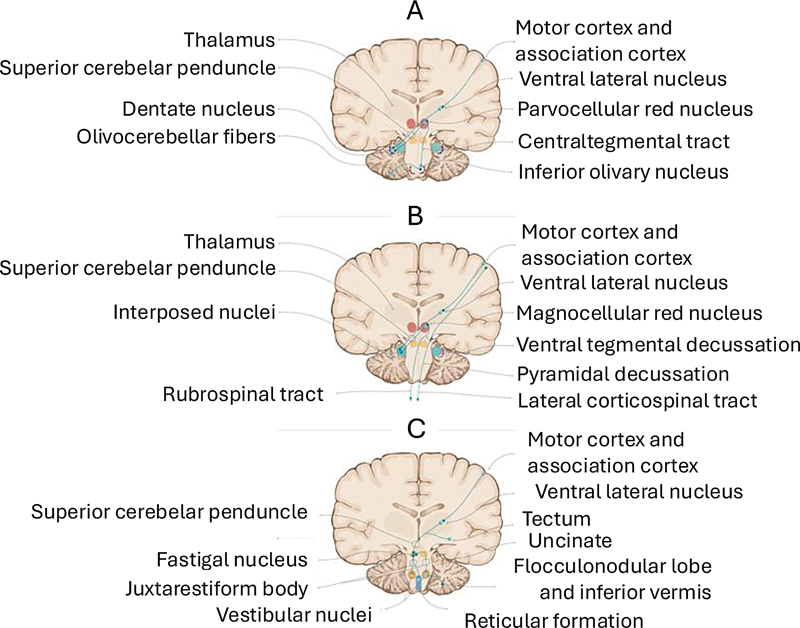
Cerebellar output pathways.

Second, the dentato-rubro-olivary pathway: projections from the dentate nucleus synapse in the parvocellular portion of the red nucleus (located in the midbrain). From there, axons project to the inferior olivary nucleus via the central tegmental tract ( Figure 3A ).

The cerebrocerebellum controls movement as a whole, including its initiation, timing, and velocity. Lesions in this region primarily result in appendicular ataxia, characterized by a delay in movement onset. This can lead to clinical signs like movement decomposition, hypotonia, dysdiadochokinesia, rebound phenomenon, kinetic tremor, and dysmetria. 5 18

The intermediate part of the cerebellum is known as the spinocerebellum. It contains the interposed and fastigial nuclei. From the interposed nucleus, projections are sent to the ventrolateral thalamic nucleus, which connects to the same areas previously mentioned in the dentato-thalamo-cortical pathway. These connections are also responsible for motor control, specifically acting on the lateral corticospinal tract. Axons from the interposed nucleus also project to the rostral portion of the red nucleus, known as the magnocellular part, which gives rise to the rubrospinal tract. In summary, the intermediate part of the cerebellum plays a fundamental role in the lateral spinal pathways, thereby acting on distal musculature and the execution of fine motor movements.

Within the spinocerebellum, the most medial portion, known as the cerebellar vermis, contains the fastigial nuclei. The projections from the fastigial nucleus exit through the superior and inferior cerebellar peduncles and are named the uncinate fasciculus and the juxtarestiform body, respectively ( Figure 3B ). Through these pathways, there are connections with four important structures: the ventrolateral thalamus, vestibular nuclei, tectal area, and reticular formation. It is essential to understand these connections, as the spinocerebellum modulates information directly within the anterior corticospinal tract, tectospinal tract, reticulospinal tract, and vestibulospinal tracts, thereby contributing to the control of proximal and axial musculature. Lesions in these pathways may result in disturbances of posture, balance, and speech, leading to truncal ataxia. Clinical signs include a wide-based, “drunk-like” gait pattern, postural instability, and dysarthria, particularly characterized by scanning speech. It is also important to note that lesions in the cerebellar vermis, due to its close relationship with the flocculonodular lobe, may lead to hypermetric saccades. 19

**Figure 4:**
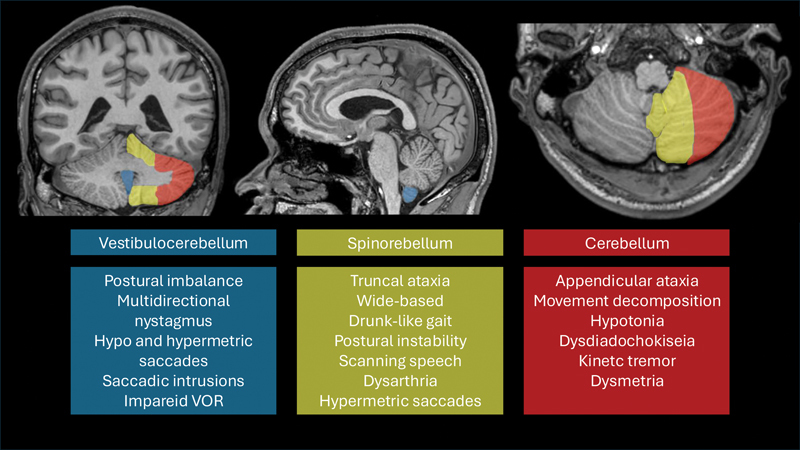
Clinical correlates of specific cerebellar lesions.

The vestibulocerebellum is represented by the flocculonodular lobe. Many authors also consider the inferior part of the vermis to be part of the vestibulocerebellum, due to its anatomical connection with the flocculonodular lobe. Lesions in this region lead to disturbances in saccadic eye movements, while lesions in the flocculonodular lobe alter posture and gait. Therefore, both structures together form the vestibulocerebellum. Projections from the vestibulocerebellum primarily target the vestibular nuclei and subsequently connect with the medial longitudinal fasciculus, an essential pathway for the vestibulo-ocular reflex and saccadic eye movements. It is important to emphasize that connections with the vestibular nuclei are reciprocal, both afferent and efferent, highlighting the crucial role of this cerebellar region in balance control. Lesions of the vestibulocerebellum result in impaired control of gait and upright posture, as well as a loss of ocular motor control during head rotation or smooth pursuit of moving targets. Patients typically present with a wide-based gait, inability to perform tandem walking, and postural imbalance. Oculomotor abnormalities are also common and may include multidirectional nystagmus, both hypo and hypermetric saccades, saccadic intrusions, and an impaired vestibulo-ocular reflex (VOR), as shown in Figure 4 . 18

## CEREBELLAR INPUT PATHWAYS

The pathways that reach the cerebellum originate from numerous regions within the central and peripheral nervous systems, representing different modalities and arriving either simultaneously or at distinct time points. For example, special sensory pathways, such as vision and hearing, reach the cerebellum via cortical pathways and via the brainstem nuclei simultaneously. 20

**Figure 5:**
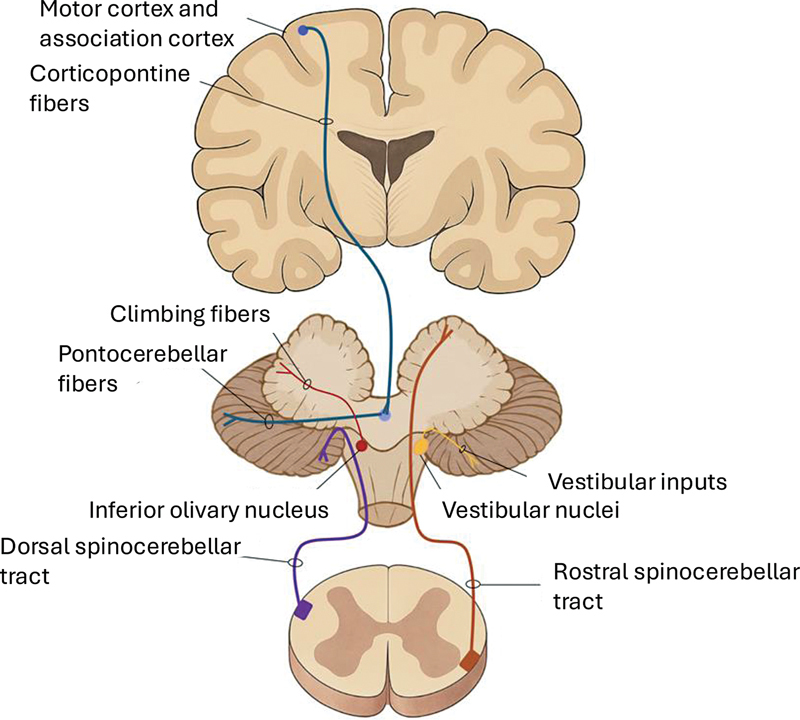
Cerebellar input pathways.

The afferent pathways to the cerebellum may originate directly from the cerebral cortex, brainstem, spinal cord, and other sensory modalities that do not necessarily require exclusive connections with these three regions ( Figure 5 ). Upon reaching the cerebellum, these inputs are directed to specific topographic functional areas.

As previously mentioned, the cerebellum shares similarities with the cerebrum in both architecture and function. Another similarity is the topographic representation of different body parts within the cerebellum. Although not as detailed as Penfield's homunculus, this organization holds significant anatomical importance. Notably, the vermis and paravermian region have representation axial body structures, while visual and auditory cortices are represented at the center of the vermis. Moving laterally within the vermis and paravermian region, body representations follow an orderly arrangement: head, arms, and legs from medial to lateral.

Within the internal cerebellar circuitry, two key structures were previously mentioned as conduits for external information: mossy and climbing fibers. These form the tracts that enter the cerebellum. Importantly, except for the inferior olivary nucleus, which gives rise to climbing fibers, all other afferent information is carried by mossy fibers. Both fiber types project to the cerebellar cortex.

The afferent pathways to the cerebellum are categorized in corticopontine, spinocerebellar, inferior olivary nucleus, and vestibular nucleus pathways. The corticopontine pathways primarily originate from the frontal, occipital, temporal, and parietal lobes. These fibers arise from the cerebral cortex, synapse at the pontine nuclei located at the base of the pons and give rise to pontocerebellar fibers, which decussate at the midline and enter the cerebellum via the middle cerebellar peduncle, projecting to its lateral regions. Interestingly, this pathway contains more fibers than the corticospinal tract. 5 9

The peripheral pathways that project to the cerebellum primarily connect through the spinal cord, with particular emphasis on the anterior and posterior spinocerebellar tracts, which enter the cerebellum via the superior and inferior cerebellar peduncles, respectively. These tracts convey proprioceptive, pressure, and tactile information from the trunk and extremities. Additionally, they transmit input from interneurons located in the spinal gray matter, which, in turn, relay information from the corticospinal tract.

Other important afferent pathways to the cerebellum include the olivo- and vestibulocerebellar fibers. A common characteristic of these pathways is their involvement in a loop, where they receive both direct and indirect input from the cerebellum. For example, the inferior olivary nucleus receives input from the red nucleus, which itself is influenced by projections from the dentate nucleus. However, it is important to note that other pathways, including cortical and spinal inputs, also contribute to this complex system by projecting to both the inferior olivary and vestibular nuclei.

The inferior olivary nuclear complex plays a key role in the integration of motor coordination, control, and learning, while the vestibular nuclei establish direct connections with the flocculonodular lobe, which is primarily responsible for balance control and vestibulo-ocular reflexes.

## CEREBELLUM AND COGNITIVE FUNCTION

The cognitive function of the cerebellum has been the subject of studies worldwide, especially with the discovery of new cerebellar neurodegenerative diseases each year. 21 22 It is well established that, both topographically and physiologically, the anteromedial regions of the cerebellum are primarily responsible for motor control, and lesions in these areas lead to ataxia.

Referring to the cerebellar homunculus, the more posterolateral regions are not associated with specific body parts. The inferior vermian/paravermian region is connected to cortical areas related to vision and hearing, as well as to the flocculonodular lobe's pathways. Based on this topographical organization and recognizing that the primary cerebral cortex connections occur within the cerebrocerebellum—either via corticopontine pathways or cerebellar efferents through the dentato-thalamo-cortical tract, in addition to climbing fibers originating from the inferior olivary nucleus—it is primarily the posterolateral cerebellum and the inferior vermian/paravermian regions that, when affected, have the most significant impact on cognitive functions. 22

The most affected cognitive domains include executive function, visuospatial abilities, language, and personality changes. Cerebellar lesions in the posterior lobe lead to deficits in executive functions similar to those observed in prefrontal cortex lesions, including impairments in planning, sequencing, verbal fluency, working memory, abstract reasoning, problem-solving, cognitive flexibility, multitasking, and organizational skills.

Lesions in the inferior vermis can result in personality changes, primarily because some cerebellar efferent pathways, such as those from the fastigial and dentate nuclei, connect to paralimbic structures, including the tectal area, hypothalamus, prefrontal cortex, posterior parietal cortex, superior temporal cortex, and parahippocampal areas, all of which are involved in cognition-emotion integration. Lesions affecting the inferior vermian and paravermian regions may lead to inappropriate behavior, mood disturbances, and psychiatric disorders.

A well-recognized syndrome associated with cerebellar-related cognitive alterations is the cerebellar cognitive affective syndrome (CCAS), first described in 1998 by Schmahmann and Sherman. They followed 20 patients over a 7-year period at the Massachusetts General Hospital, United States. It was possible to correlate bedside neurological examination findings with neuropsychological assessments, which consistently matched anatomical localization, neurophysiology, and functional imaging studies. 23 24 In summary, CCAS is characterized by alterations in executive functions, including poor planning, difficulty with set-shifting, impaired abstract reasoning, working memory deficits, and reduced verbal fluency. It also includes spatial cognitive impairment, such as visuospatial disorganization and visuospatial memory deficits. Personality changes are common, typically presenting as affective flattening or blunting, along with disinhibited or inappropriate behaviors. Language difficulties may also be present, including dysprosody, agrammatism, and mild anomia.

In conclusion, the cerebellum, historically recognized for its role in motor control and movement coordination, has emerged as a neurophysiologically complex structure with functions that extend far beyond motor regulation. The discovery that the cerebellum actively participates in circuits related to cognition and emotional control has gained increasing attention in research each year. Understanding cerebellar neurophysiology is of utmost importance in clinical practice, as the cerebellum serves as a key integrative center within the central nervous system. Given the presence of distinct cellular layers, zones, circuits, afferent and efferent connections, deep nuclei, neurons, glial cell types, and lobes, it is essential to correlate not only its anatomy but also its pathophysiology and apply this knowledge to various cerebellar disorders.

Neurovascular diseases, tumors, neurodegenerative disorders, and genetic conditions are some examples that can lead to ataxias and neuropsychiatric disturbances. Therefore, a comprehensive understanding of cerebellar neurophysiology is crucial, not only for the differential diagnosis of cerebellar syndromes but also for guiding therapeutic and rehabilitative strategies. This knowledge enables the development of more precise interventions that address both motor deficits and cognitive dysfunctions resulting from cerebellar injury or dysfunction.
